# Diet in Acne Vulgaris: Open or Solved Problem?

**Published:** 2017-03

**Authors:** Jakub MORZE, Katarzyna Eufemia PRZYBYLOWICZ, Anna DANIELEWICZ, Małgorzata OBARA-GOLEBIOWSKA

**Affiliations:** 1. Dept. of Human Nutrition, Faculty of Food Sciences, University of Warmia and Mazury in Olsztyn, Olsztyn, Warmian-Masurian Voivodeship, Poland; 2. Dept. of Psychology of Development and Education, Faculty of Social Sciences, University of Warmia and Mazury in Olsztyn, Olsztyn, Warmian-Masurian Voivodeship, Poland

## Dear Editor-in-Chief

Acne vulgaris is one of the most common dermatoses, which occurs adolescents and increasingly young adults (1). It is known to impair many aspects of quality of life. Acne vulgaris leads to poor body image, anxiety, depression, anger, frustration, diminished self-esteem, social isolation and restriction of activities (2). Over the years, two approaches to investigating acne were established: dermatological and epidemiological. Considering the multifactorial base, chronicity and demographic character of the acne, it could be qualified as a non-communicable disease. One of the fields of public health research is analysis of the dependence between NCDs and exposome (complement of environmental conditions), looking for modifiable risk factors (3). Irrefutably, the key role is played by diet and nutribehaviour.

The cross-sectional study was conducted on 370 students aged from 14 to 19 yr (Mean=16.0±1.5), both healthy and suffering from acne vulgaris in 2015. The legal basis of the research was a permission of the UWM FOMS Bioethical Committee (No. 9/2015).

To obtain data about diet validated *Food Frequency Questionnaire with six answers* (FFQ-6) was used. The questionnaire consisted of 68 products, divided into 8 main groups of food: sweets and snacks, dairy products and eggs, cereal products, fats, fruits, vegetables and seeds, meat and fishes and beverages. Additionally, questions about caffeinated coffee, non-caffeinated coffee, black tea, green tea, and spices consumption were affixed. The acne intensity was examined using *The Leeds Revised Acne Grading System* – the facial lesions by dermatologist, the back and chest self by contributors after showing them the scale key.

The statistical analysis was provided with Statistica 12.0 PL software. Collected data was used in PCA to identify dietary patterns. Subsequently, using a Kruskal-Wallis ANOVA, differences of acne intensity grades in terciles of dietary patterns were compared. The statistical significance level was 0.05.

**Fig. 1:**
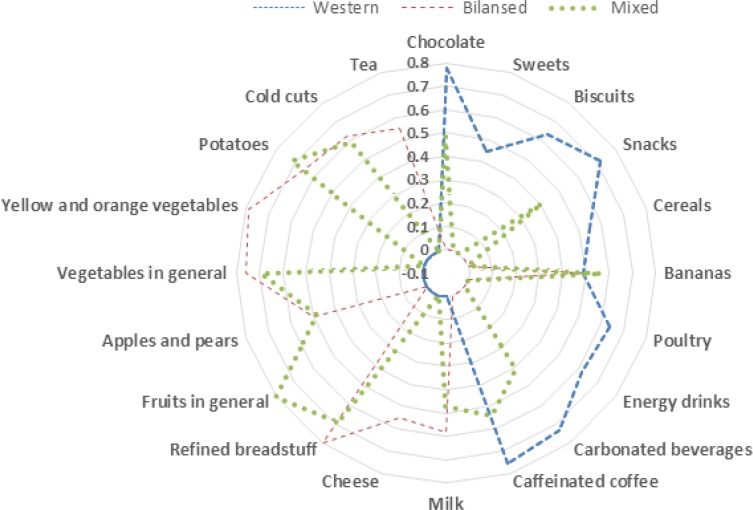
Rotated factor loadings of identified dietary patterns (Western, Bilansed and Mixed) of food groups retained. Not shown are food items with values less than 0.4

Three dietary patterns were extracted that best describe the patterns of food intake in participating group. 42.3% of the variability was explained. The total explained variability amounted to 45.1%. The first explaining 25.6% was followed by 13.1% and 6.4% (Fig. 1). Significant differences of acne intensity (Table 1) was observed in terciles of “Western” (Face lesions *P*=0.03) and “Mixed” (Face lesions *P*=0.04 and Back lesions *P*=0.02).

**Table 1:** Association of dietary patterns with acne intensity on face, back and chest

**Dietary pattern**	**Tercile**	**Face lesions**		**Back lesions**		**Chest lesions**	
		**Mean**	**95% CI**	**Mean**	**95% CI**	**Mean**	**95% CI**
**Western**	**1** <0.36; 2.55)	1.54	(1.49; 1.60)	0.35	(0.24; 0.45)	0.17	(0.09; 0.24)
	**2** <2.55; 4.07)	1.72	(1.60; 1.85)	0.49	(0.38; 0.60)	0.22	(0.14; 0.31)
	**3** <4.07; 15.93>	1.89	(1.63; 2.15)	0.44	(0.32; 0.56)	0.25	(0.16; 0.35)
***P***			0.03		0.15		0.36
**Balanced**	**1** <0.45; 2.04)	1.70	(1.47; 1.93)	0.49	(0.36; 0.62)	0.24	(0.14; 0.33)
	**2** <2.04; 2.87)	1.73	(1.47; 1.99)	0.40	(0.30; 0.51)	0.22	(0.14; 0.30)
	3 <2.87; 10.21>	1.72	(1.48; 1.96)	0.38	(0.27; 0.49)	0.18	(0.10; 0.26)
***P***			0.19		0.52		0.55
**Mixed**	**1** <0.48; 1.80)	1.57	(1.34; 1.80)	0.37	(0.26; 0.48)	0.23	(0.14; 0.32)
	**2** <1.80; 2.56)	1.71	(1.47; 1.95)	0.37	(0.27; 0.47)	0.21	(0.13; 0.29)
	**3** <2.56; 7.95>	1.87	(1.63; 2.11)	0.54	(0.49; 0.59)	0.20	(0.12; 0.28)
***P***			0.04		0.02		0.81

Previous theoretical studies pointed about several aspects that allow linking diet and acne. These are high glycemic index, high glycemic load diet, insulin/insulin-like growth factor 1 (4), and androgen sebocyte stimulation. Majority of available research articles dealing with the effect of nutrition on acne concentrates on the impact of single dietary factors usually connected to particular etiological mechanism (5). However, there are several limitations of these works. Only a small proportion of the available food items are considered. Most frequently described food groups are sweets, junk food, snacks, milk, and chocolate. In our study, additional products such as coffee, tea, potatoes, vegetables and fruits in general, as well as refined breadstuff were introduced. Several of them never or very rarely were mentioned in literature. Besides, not all of them refer to western-like diet, on which many authors focus the most. Moreover, singular analyses are very sensitive to the distribution changes and lack of multivariable approach may lead to wrong reasoning. Of course, conducting a few high-quality studies would not solve the problem, because obviously, they cannot be basis to create dietary recommendations, but they are invaluable guidelines for researchers.

Generally, establishing consistent nutriepidemiology and prophylaxis in acne is a real perspective. Patients increasingly begin to understand acne more as a social obstacle, than medical condition. Acne lesions are serious impediment for young people, who more and more pay attention to their own appearance to conclude, issue of diet impact in acne is still an open problem. This study pointed out several limitations of foregoing works, which should be straightened up. We hope that this is the outset to further investigations, which would lead us to solution of the problem.
